# The Role of Networks in Mobilization for Ethnic Minority Interest Parties

**DOI:** 10.1007/s11109-024-09988-w

**Published:** 2024-11-09

**Authors:** Rutger Schaaf, Simon Otjes, Niels Spierings

**Affiliations:** 1 Institute of Political Science, Universiteit Leiden, Wassenaarseweg 52, Leiden, 2333 AK The Netherlands; 2 Radboud Social Cultural Research, Radboud Universiteit, Houtlaan 4, Nijmegen, 6525 XZ The Netherlands

**Keywords:** Ethnic-Minority Interest Party, Voting, Muslims, Social Networks

## Abstract

Recently, parties that are run by and for ethnic minority citizens with a migration background have become more prominent. They can be considered a manifestation of ethnic political segregation. A key example of such a party is DENK in the Netherlands. So far, the explanatory literature has focused on how programmatic considerations drives voting for these parties. Other factors, such as the role of social networks in mobilization, have received limited testing and limited exploration in more detail. Furthermore, the literature on social networks is mainly based on majority populations. To inform our understanding of the role of social networks in voting (in general but also particularly among ethnic minority communities and for ethnic minority interest parties) this paper analyzes the voting behavior for DENK focusing on the role of personal, online and religious networks. The paper uses both qualitative interviews (with bicultural youth in the third largest city of the Netherlands in 2022) and quantitative surveys (the 2021 Dutch Ethnic Minority Electoral Study). Our analysis points to the importance of religious and personal networks for voting for DENK, whereas online networks appear to be less relevant.

**Supplementary Information:**

The online version contains supplementary material available at 10.1007/s11109-024-09988-w.

## Introduction

Across Western democracies, ethnicized and racialized minorities citizens feel underrepresented, evaluate politics more negatively than majority citizens, and are shown to turn out less (De Vroome et al., 2013; Heath et al., 2013; Spierings & Vermeulen, 2023; Stevens and Bishin, 2011; but see Dupree and Hibbing, 2022). The political behavior of this growing electorate (Spies et al., 2020) can however make a distinct impact on election outcomes, swaying the results in majoritarian systems and leading to fielding minority candidates (Banducci et al., 2004; Farrer et al., 2018; Sadhwani, 2022). In proportional systems power relations might change particularly by strengthening the position of minority candidates (Azabar et al., 2020; Bergh & Bjørklund, 2011; Van der Zwan et al., 2020) and weakening the position of mainstream left-wing parties when minority voters turn to new parties (Lubbers et al., 2023; Otjes & Krouwel, 2019).

Ethnic minority interest parties are a new party family. Such parties have been participating electorally for about a decade now and are increasingly successful. They have run in elections in Austria, Belgium, Denmark, Germany, the Netherlands, the UK, and Sweden, winning local seats in multiple countries. In 2017, DENK was the first of these parties to win multiple seats in a national parliament, in the Netherlands (Loukili, 2021; Lubbers et al., 2023). The recent rise of ethnic minority interest parties is, however, understood poorly, while understanding their support is an important case for understanding the larger issue of how the political behavior of ethnicized and racialized minorities shapes politics across Western democracies and political systems.

The small existing body of literature trying to understand who votes for ethnic minority interest parties (and who does not) mainly focused on policy positions and ethnicity- and discrimination-related distrust as explanations (Otjes & Krouwel, 2019; Van der Zwan et al., 2020; Vermeulen et al., 2020a). In this study, we extend this literature by bringing in (and contributing to) the literatures on social networks in vote choice and social capital in participation.

Social network literature has been applied to voting, but the importance of social networks has not been applied to voting for ethnic minority interest parties, at least not beyond indirect references.^1^ Social networks and voting studies more generally, however, underpin that political preferences are updated later in life, partially through networks (Cho et al., 2006; Lazarsfeld et al., 1944; White and Laird, 2020). Santoro and Beck (2017, p.386) stress that nearly all work in this field focuses on the U.S. and that little attention is given to how the impact of social networks is interlinked with voters “enduring personal characteristics”, such as ethnic and racial background. From the literature on social integration and social capital we know social surrounding is an important driver of political behavior, particularly among ethnic minorities (Banducci et al., 2004; Bird et al., 2010; Fennema & Tillie, 1999; Lubbers et al., 2023; Moutselos, 2020; Schrover & Vermeulen, 2005; Spierings & Vermeulen, 2023; Stevens and Bishin, 2011; White and Laird, 2020), but this literature either does not have a focus on vote *choice* or takes a narrow focus on a particular network aspect.

Across these literatures, we argue – as is the case for other political and social behavior (Spies et al., 2020) – that it is unknown if and how a logic based on studies on the majority population translates to ethnic minorities. So, we posit that integration in social networks may steer ethnic minority people’s vote, particularly for ethnic minority interest parties. There is still much unclarity as to how and which aspects of social networks matter, as conclusions on the study of majorities cannot be simply generalized. Applied to our case (support for the ethnic minority interest party DENK) we thus set out to answer the following research question: *to what extent and under what conditions does the integration in various social networks of ethnic minority citizens affect their likelihood to vote for ethnic minority interest parties?*

We answer this question using a mixed-methods design: we first employ 16 in-depth interviews with Moroccan-Dutch and Turkish-Dutch voters. Combining these interviews and the existing literature, we distil three distinct, albeit interrelated, factors that seem to be plausible social network influencers of voting for ethnic minority interest parties: religious institutions, social contacts, and online networks. Next, these factors are translated to five specific hypotheses that we test empirically using the Dutch Ethnic Minority Elections Study (Lubbers et al., 2022; Sipma et al., 2021), which includes data on all these factors.

Altogether, this study’s contributions relate to two larger questions on political behavior: (1) what (partially unique) processes shape the political behavior of ethnicized and racialized groups, groups that are becoming numerically and discursively more important in Western democracies given demographic changes and the rise of exclusionary discourses, and (2) how do social network theories apply to ethnic minority voters and what new insights on such a mechanism derived from this focus might enrich the larger literature.

## Theory

Our focus on the importance of social networks starts from the larger literature on social capital and social networks. To provide a theoretical lens by which we analyze our interviews, we distinguish three aspects of social capital: organizational, personal and digital.

### Organizational integration

Social capital is often interpreted as integration into intermediary social organizations, for instance by visiting religious meetings or volunteering in organizations. This leads to strong social networks and consequently spurs participation in politics, such as voting. This effect has been observed particularly among members of ethnic minorities and migrant communities (Bird et al., 2010; Schrover & Vermeulen, 2005; Zuckerman et al., 1994) and has been linked to several mechanisms which can be translated to vote choice.

Activity in religious organizations brings ethno-religious minorities knowledge of the political system (Moutselos, 2020; Oskooii and Dana, 2018). This feeds into political interest and internal efficacy (Calhoun-Brown, 1996; Spierings & Geurts, 2023; Sobolewska et al., 2015). As more knowledgeable voters are more likely to switch parties (Pharr and Putnam, 2000), organizational integration might increase the likelihood of voting for newer ethnic minority interest parties.

A second mechanism focuses on group identities and grievance. As ethno-religious organizations are organized around a common, marginalized identity, shared grievances may become salient via organization (Ben-Nun Bloom and Arikan, 2012). In turn, religious organizations foster stronger in-group bonds (Uslaner, 2002; Zuckerman et al., 1994). Moreover, in the context of perceived group discrimination, in-group ties may strengthen. This in-group focus is significant because it likely influences voting for ethnic minority interest parties through two steps: first, voting serves as a means to address the sources of grievances; and second, when individuals’ attention is centered on the in-group, they may be inclined to support a party that represents and advocates for ethnic minorities, distancing themselves from parties primarily associated with the ‘out-group.’

Finally, parties and politicians actively use ethno-religious organizations for political mobilization and campaigning (Pries & Sezgin, 2012; Schrover & Vermeulen, 2005). Indeed, religious networks provide recruitment networks and resources that political actors use in order to campaign for their party. This not only mobilizes turnout (Moutselos, 2020), but, assuming the campaigning is at least to some extent effective, also directs that vote.

All in all, it is plausible that integration in ethno-religious organizations can increase the likelihood of voting for an ethnic minority interest party. Other work does suggest it is not that simple: for instance, Azabar et al. (2020) found no impact of mosque attendance on the likelihood casting a preference vote for a Muslim candidate, and Kranendonk and Vermeulen (2019) show that the in-group network building can decrease turnout. The current literature thus warrants a focus on ethno-religious organizations, offers some likely mechanisms at play, but also raises questions about the linkage between integration in these kinds of organizations and voting.

### Personal social networks

Personal networks partially work in similar ways as described above: the core focus is on having ties with people who are ethno-religiously similar. For instance, neighborhood studies have argued that, in neighborhoods with more ethnic minority citizens, social contacts between members of these communities are an important source of political knowledge and influence political behavior, including voting (Cho et al., 2006; Vermeulen et al., 2020b). Group norms matter here, as White and Laird (2020) show for Black Americans. Likewise, Van der Zwan et al. (2020); Vermeulen et al. (2020a) argue that the prevalence of ethnic minority preference voting increases disproportionally the great the minority is a neighborhood, although there are indications such effects vary by community (Vermeulen et al., 2020b).

More generally, it has been shown that input from political campaigns and the preferences of one’s social ties provide cues for updating one’s views and preferences, and this input can come from close and strong ties, as well as weaker ones (Lazarsfeld et al., 1944; McClurg et al., 2017; Wood, 2000). In other words, voting preference is not only based on clearly set contexts like formal organizations, but neighborhoods and social ties also matter. Bringing this together with the literature discussed above suggests that having more social ties with other ethnic minority citizens might heighten the chance of voting for an ethnic minority interest party, whereby rather similar mechanisms are implied as for ethno-religious organizations.

### Virtual networks

Finally, while not featuring in the discussions above, our more general perspective on the role of social networks cannot ignore the increased importance of social media and digital networks in politics, particularly in an early-adopter, proportional system such as the Netherlands (Jacobs and Spierings, 2014).

Increasingly, parties use social media to mobilize their voters; candidates use it to gain personal votes (Kligler-Vilenchik et al., 2021). Social media offers unique opportunities to lower-ranked and marginalized politicians, for whom social media create ‘third spaces’ (Jacobs and Spierings, 2016). Here, minority politicians and parties use their voice to address their concerns without interference from gatekeepers (Loukili, 2021). Online networks are not wholly separate from offline networks; they likely activate existing social and political identities and interests.

Citizens can get in contact with individual politicians via social media and relate to the people behind the politicians, with less control from the media and party leadership as gatekeepers than what is seen in offline spaces (Jacobs and Spierings, 2016). Citizens also come across political (re)posts more often, and this information may reach them via friends and acquaintances, whom they may trust more than any individual politician. Ethnic minority citizens who are more politically active on social media may be enticed to vote for ethnic minority interest parties, and build on the digital, personal networks of ethnic minority citizens (Loukili, 2020, 2021).

We conduct our qualitative analyses in response to this interpretation of the literature, integrating the results to develop more specific hypotheses, which are subsequently tested.

### Case description

We study DENK as the most prominent and most stable ethnic minority interest party in the Netherlands (Lubbers et al., 2023). The Netherlands has a complex multiparty landscape, and the electoral system allows for the representation of all kinds of parties. However, ethnic minority interest parties, i.e., parties run by and for citizens with a migration background do exist in other West European countries, and sometimes win (sub)national representation (Lubbers et al., 2023).

DENK was formed in 2015. Two Turkish-Dutch MPs, Kuzu and Öztürk, left the Labor Party after a conflict with the Labor minister responsible for the civic integration of minorities. They participated in the 2017 elections under the name DENK. This name means ‘Think’ in Dutch, and ‘Equal’ in Turkish. The party won three seats in the national parliament, and they consolidated this in subsequent national elections. In the 2018 municipal election, they gained seats in thirteen municipal councils, and in 2022 municipal election, they also won seats in the city council of The Hague.

In the Netherlands, the Turkish-Dutch community is well-organized (Michon & Vermeulen, 2013; Fennema & Tillie, 1999). Scholars have emphasized DENK’s strong ties to this community (Vermeulen and Kranendonk, 2019). Their candidate list, however, also includes people with other ethnic backgrounds, most particularly Moroccan-Dutch and Surinamese-Dutch citizens (Lubbers et al., 2023). In 2021, their party leader was the Moroccan-Dutch Azarkan.

DENK’s manifesto offers a mix of progressive positions on questions related to immigration, centre-left positions on economic matters, and conservative positions on moral issues. Its manifesto is oriented at fighting discrimination and Islamophobia and includes specific measures to protect the civic spaces of Islamic, Turkish-Dutch and Moroccan-Dutch communities from targeted government interference and extremist violence and harassment. This includes protecting the right to found Islamic schools and the right to ritual slaughter. Other topics include banning demonstrations in front of religious buildings, ending the ban on the burqa in public spaces and ending the requirement for Turkish migrants to take integration courses. Moreover, they argue for more government spending on mosque protection against extremist violence and for detectors to automatically track down incitements of hatred on social media.

## Qualitative analysis

We use qualitative interviews to explore the tenability of the relationships sketched above, and to refine the mechanisms that might matter.

### Qualitative methodology

We held sixteen semi-structured interviews with members of the Turkish-Dutch (8) and Moroccan-Dutch (8) communities. These are the two of the largest migrant communities in the Netherlands. We interviewed respondents who have voted for DENK as well as those who have not, which helps to identify under what conditions the target population *does* and does *not* vote for DENK. We held interviews in the weeks after the 2022 municipal elections. Given that DENK does better among younger voters than among older voters (Otjes & Krouwel, 2019), our interviews focused on people aged between 20 and 30 years (average: 23; standard deviation: 3). Seven respondents were women and nine were men. All respondents were students, but at different institutes: two respondents studied at a vocational college, six at polytechnical institutes and eight at a research university. Eight respondents voted DENK in the national elections and five in the municipal elections. All respondents identified as Muslim. Table A1 in the Appendix lists more information on the interviewees. We held the interviews in The Hague. This is the third-largest city of the Netherlands with a diverse, though segregated population. DENK scored 6% in the municipal elections. Nationwide, DENK scored more than a third of the vote in 20 polling stations during the 2022 municipal elections; nine of these were in The Hague.

The respondents consist of a varied group of voters in terms of voting behavior, educational background, ethnicity and gender. We are aware that this group is not a representative sample of either the migrant electorate or the DENK constituency. We contacted students at educational institutions in The Hague: student associations helped to spread a call for interviews among their members and one of us^2^ posted at the educational institutions and asked students directly to participate in an interview. We pursued this strategy and target group because the Turkish-Dutch and Moroccan-Dutch communities are increasingly politically alienated (Vermeulen and Kranendonk, 2019) and can be closed off for researchers. Given this numerical shortcoming, we have only used the interviews to give us insights into the mechanisms that might matter for DENK voters, from which we can derive expectations that we then test on a representative sample of the migrant population.

Appendix A1 shows our interview guide. During the semi-structured interview, participants were given room to mention other factors that influenced their voting behavior and were informed that the interviews were held in the context of a larger project on ethnic minority political participation.

We recorded the interviews, transcribed them and coded them for analysis. We employed Burnard’s (1991) abductive method for thematic content analysis of interview transcripts. This involved initial open coding guided by the study’s focus to understand the variety of processes at play across and within the interviews. We aggregated coded material in the same category for each respondent, and then divided them according to those that did and did not vote DENK and according to those from Moroccan-Dutch and Turkish-Dutch voters. In these different categories, we tried to find general patterns per group, using the social pattern analysis (Zerubavel, 2007). This is a method to find general patterns in data from different cultural contexts. Appendix A1 lists the coding scheme and Table A2 the final patterns. The quotes were translated from Dutch by the authors.

### Ethno-religious organizations

We asked to what extent politics was discussed in mosques and non-religious migrant organizations, and to what extent political parties were present to campaign there.

In Turkish-Dutch mosques, politics is an important subject of debate. As one Turkish-Dutch respondent stated, “Turks speak about politics everywhere”. However, when Turkish-Dutch people speak about politics, they focus on Turkish politics and only discuss Dutch politics “when it comes down to it”. Still, multiple Turkish-Dutch respondents indicated that they had voted for local DENK candidates that they knew through attending the mosque. One respondent had met DENK MP Kuzu at a mosque in The Hague: “Friday prayers are of course always very busy. And then he comes to speak to the Turkish community. He speaks in Turkish and shares his ideas.” Another Turkish-Dutch respondent told us that politicians of DENK and other parties went around Turkish-Dutch mosques, associations, and sport clubs to talk to their constituents. The interviews showed the mosque to be a space to discuss politics, but the imam in Turkish-Dutch mosques appears to stay politically neutral and does not campaign for a party himself. While the above includes traces of the different mechanisms from existing literature, the campaigning mechanism linking the integration in religious organization to voting for ethnic minority interest party seems strongest.

This was markedly different for the Moroccan-Dutch respondents, who all emphasized that politics is not discussed in the mosque, a politically neutral sphere. In the words of a Moroccan-Dutch respondent who voted DENK: “The mosque is a place where politics is not discussed. That has to do with the history of Morocco. […] politics in the mosque, that is a no go.” Moroccan mosques are also not a place where political parties campaign, since “it really is a house of prayer”. However, that does not mean that political parties do not campaign *close to* the mosque. Parties have signs outside of the mosque and, during the campaign, party activists are present on the street next to the mosque after Friday prayer. As a Moroccan-Dutch respondent described: “So, on Friday I go to the mosque to pray and when I come outside, then all political parties are present hunting for people […] in front of the mosque […]. ‘Here, have this bag of gifts’ or ‘here, a leaflet’.” Some Moroccan-Dutch respondents were also active in non-religious migrant organizations. Here, political issues were discussed, but political parties were not involved, and the organizations try to remain politically neutral give the diversity of political preferences in the Moroccan-Dutch community; they only try to convince people to turn out to vote, without supporting a specific party. So, while the mechanisms seem weaker, mosque attendees are exposed to ethnic minority interest parties’ campaigns, but little evidence for the other mechanisms is found.

Overall, for both Turkish-Dutch and Moroccan-Dutch respondents, the mosque, and, to a lesser degree, other organizations, are a site of campaigning, particularly for DENK. However, this is much stronger in the Turkish community than in the Moroccan community. The interviews illustrate a mechanism that aligns with the ‘campaigning’ mechanism, but in a specific manifestation. We found no signs that there were direct calls from the mosque organizations to vote for a certain party, but DENK was visibly campaigning around mosques. No direct traces were found for the other mechanisms, but we do see initial links that suggest they might still matter. Regarding the ‘knowledge transfer’ mechanism, voters did not talk about a direct transfer through mosque leadership for instance, but particularly for the Turkish-Dutch respondents the mosque is a site of political discussion, which in the literature is shown as a core way to transfer knowledge. Similarly, considering the DENK manifesto, and its campaign near the mosque, it may make group grievances more salient, suggesting that so-named mechanisms still matter. Altogether, the interviews provide sufficient support for us to propose:

1. *Religious Network Hypothesis*: The more often citizens attend a mosque, the higher their propensity to vote for an ethnic minority interest party.
2. *Turkish-Dutch Religious Network Hypothesis*: The effect of mosque attendance on voting is stronger for the Turkish-Dutch than the Moroccan-Dutch citizens.

### Personal networks

Respondents themselves brought up how important personal contacts were to their vote choice. Not only does this refer to respondents knowing local candidates personally, but also the importance of family members, particularly when respondents themselves were not interested in politics. As one Moroccan-Dutch DENK voter said: “politics is an issue in our home. It is discussed very often. In particular, my brother and my father, who are really strongly engaged […]. [M]y brother briefly explains what the party manifestos say, and he leaves me free to make my own choice.” Multiple respondents noted that they followed the preferences of their family members more explicitly. As one Turkish-Dutch respondent said: “[I followed] the advice of my father […] He knows more about politics than I do, I hardly watch politics.” A Turkish-Dutch respondent emphasized how he influenced his mother to vote DENK: “My mother follows what I say […]. I can convince her, but my father is more difficult […]. [H]e follows the discussions.”

Overall, the in-depth interviews support the idea that personal contacts affect vote choice. Voting for an ethnic minority interest party seems to benefit in particular, not because it is the only party supported in the personal network, but because it is a relatively new party that gets less attention in regular media, which thus has more to win from these networks. We learn from the interviews how important it is for information to come from a trusted, familial source who is considered knowledgeable. Therefore, we propose:

3. *Personal Network Hypothesis*: The more people in the personal network of citizens with a migration background vote for an ethnic minority interest party, the higher the citizens’ propensity to vote for an ethnic minority interest party.
4. *Familial Network Hypothesis*: the effect of the personal network on voting for an ethnic minority interest party is stronger if those people in the personal network are family members.

### Virtual networks and social media

Finally, we also asked respondents about online political mobilization. We asked whether they often see messages about politics online, and whether these posts come from people they know or were party advertisements and what their content was.

First, all interviewed respondents used social media. Nearly all had seen messages about politics on social media, including both party advertisements and posts from people they knew. The respondents indicated, independently of whether they voted DENK and independently of their ethnicity, that they saw messages from different parties. However, DENK voters did indicate that they often saw messages from this party, which was also because they had followed the party or members of the party on social media. As one Moroccan-Dutch DENK voter indicated: “I follow DENK on social media. And what DENK does is, if they have participated in a debate, then they share that debate on Facebook […]. And I find that interesting. Because I can see that they have proposed a motion that I tend to agree with.”

More generally, the short clips that DENK produces and shares are mentioned by different DENK voters. Often these are “catchy” movies of MPs Azarkan or Kuzu, with “a nice beat behind it, very exciting”; clips in which Kuzu “defeats” radical right-wing MP Geert Wilders “with his words”. Many of them indicated that they came across such clips via friends’ or family’s shares. One Moroccan-Dutch respondent mentioned that clips from debates often go “viral”. Indeed, respondents that did not vote for DENK also indicate that they saw these messages. One elaborated that “[p]arties like DENK […] they know well where they need to be, and they know on which Facebook pages or Instagram pages they have to tell their story. […] I am in many groups, including in Facebook pages of academic Muslim students or foreign students. You can sense that (the members of these groups) are more active for certain parties.”

In brief, the respondents often saw DENK on social media, both from trusted sources and from the party directly. These clips seem to be appreciated, and DENK may reach their electorate via online networks more than other parties. At the same time, their impact seems less obvious as non-DENK voters also come across them a lot, and DENK supporters actively follow DENK, indicating that it is their preference shaping their exposure, not the other way around. Or, from a slightly different perspective, although the party might be effective in reaching ethnic minority citizens, it may not make a significant difference to the voting preferences of ethnic minority voters. Nevertheless, the professional messages are shared – a necessary condition for online networks to have an impact – and thus we can formulate the following hypothesis in line with the literature:

5. *Online Network Hypothesis*: The more often citizens with a migration background see political messages on social media, the higher their propensity to vote for an ethnic minority interest party.

## Quantitative analysis

The literature informed our frame of understanding; the qualitative analyses deepened this and helped us to formulate more specific hypotheses. Here, we take the next step, testing these hypotheses using quantitative data.

### Data Collection

We use the Dutch Ethnic Minority Election Survey (DEMES) (Lubbers et al., 2021; Sipma et al., 2021). It was designed as a sample of Dutch citizens with a so-called ‘migration background’ that were at the time categorized as ‘non-Western’ by Statistics Netherlands, and who were eligible to vote in the 2021 elections. Statistics Netherlands used the government definition of these categories; a political construct. The sample consists of people born either outside of the Netherlands in a ‘non-Western’ country or to at least one parent born in such a country. Here, ‘non-Western’ refers to Asia,^3^ Africa, or South America. The group demarcated by these criteria make up a little over 10% of the Dutch electorate. 40% have a migration background in Morocco or Turkey.

The survey team approached the sample before the parliamentary elections of March 2021. Respondents who participated before the elections were also invited after the elections to fill out a post-election questionnaire. The surveys were self-administered online (push-to-web contact), reducing the risk of socially desirable answers, with the option to receive a self-administered paper survey.

Response rates below 20% in surveys of populations with a migration background are not unusual (Kappelhof, 2015). For DEMES, the response rate was 22% (Sipma et al., 2021). Given our push-to-web survey, this response rate is within the range of what could have been expected (Sobolewska et al., 2022). Moreover, the surveys were conducted in Dutch, but as the right to vote is linked to citizenship and thus living in the Netherlands for at least 5 uninterrupted years, the impact on non-response among voters due to language issues is likely limited. The largest issue in terms of response rate, however, is the two-wave nature of DEMES. Out of the 765 respondents, 361 did not participate in the post-election survey. Some issues are only addressed in the second wave of the survey, decreasing the N. To deal with small N, we pursued a range of strategies (see Modelling Strategy below).

### Dependent variable

As a dependent variable, we use propensity to vote (PTV): the self-reported likelihood that a voter might vote for a certain party. Specifically, respondents are asked whether they could indicate on a scale from 1 (never) to 10 (certainly) how probable it is that they would ever vote for each party.^4^ We set the option ‘I don’t know this party’ as missing.^5^

### Independent variables

To examine the (ethno-)religious network question, we can use two DEMES items. The first is the extent to which respondents attend religious services.^6^ As an alternative, we have the question whether the respondent is a member of a religious organization. This is a binary variable which comes from the second wave of the survey (with a smaller N). To assess the Turkish-Dutch Religious Network Hypothesis, we interact these with ethnic groups:^7^ the question identified whether the respondent has a Turkish, Moroccan or other Middle Eastern/North African migration background

To examine the personal networks, we examine two sets of indicators: the first set examines the ethnic makeup of the friend networks of respondents. Respondents were asked about how often they meet up with friends without a migration background and friends with the same migration background as themselves. We examine the difference between these two: the extent to which respondents are embedded in a friend network of co-ethnics or people without a migration background. The Appendix looks at these variables separately. Secondly, to test the Familial Network Hypothesis, we also examine the extent to which people in their family or friend network vote for an ethnic minority interest party. We examine these separately (for parsimony, looking at partner, parents, and friends, see model A10 in Table A5) and as a scale.^8^ This variable, however, is only asked in the second wave.

To examine the virtual networks of the respondents, we can only use one available question: the extent to which the respondent sees messages about politicians or current affairs on their social media. This question is not ideal for multiple reasons: firstly, it was only asked in the second wave; secondly, it does not distinguish between sources of the messages; thirdly, it does not concern DENK specifically. Despite this, we employ it, as it does account for social media use and, as shown in the qualitative analyses, Moroccan-Dutch and Turkish-Dutch social media users can hardly avoid DENK messages.

We control for gender,^9^ age,^10^ education level,^11^ whether a respondent is Islamic, and the difference between respondents’ left–right self-identification and their positioning of DENK.

### Modelling Strategy

We run linear regressions on the PTV data. Given the constraints of the N, in particular when using items from the second wave of the survey, we run different models. The main results are in Table 1. This presents two pairs of three models. The first three lack interactions, which the second three add. The first model only includes variables from the first wave. This lacks any indicator on social media use. The second and third have variables from both waves. These models have a lower N. The models differ in how they approach both the ethnicity of respondents’ social networks and the voting patterns of these social networks (as a scale or separately).

Appendix A2 presents 29 other models. Table A5 examines the effect of our predictors in isolation, with only the controls as additional variables. Table A6 separates the variable that looks at the ethnic makeup of the respondents’ friend group into a separate indicator for co-ethnics and respondents without a migration background. Table A7 interacts backgrounds in Morocco, Turkey and other MENA countries with the main predictors from both waves. Table A8 controls for the left-right dimension. These appendices present the same three models as the main paper. Table A9 limits the analysis to respondents with a MENA (including Moroccan and Turkish) background. To ensure a sufficient N in Table A9, we only use variables from the first wave. Table A10 compares interactions between the religious embeddedness variables and being Islamic and having a Turkish background. Table A11 interacts respondents’ personal and social media networks. Even the model with the smallest N (Table A8) does not have more predictors than its N would justify (Green 1991).

**Table 1:** Linear Regression Models

Model	1	2	3	4	5	6
Intercept	2.25^***^	2.19^***^	2.21^***^	2.43^***^	2.24^***^	2.25^***^
	(0.31)	(0.59)	(0.58)	(0.32)	(0.59)	(0.59)
Gender = Male	− 0.01	0.11	0.07	− 0.06	0.08	0.04
	(0.26)	(0.34)	(0.33)	(0.26)	(0.34)	(0.33)
Age ≤ 30	0.41	0.02	− 0.15	0.42	0.01	-0.15
	(0.27)	(0.36)	(0.35)	(0.27)	(0.36)	(0.35)
Education = BA/MA	− 0.01	− 0.03	0.07	0.02	0.01	0.09
	(0.26)	(0.34)	(0.34)	(0.26)	(0.34)	(0.34)
Country of Origin = Morocco	1.12^**^	0.52	0.24	1.26^**^	0.57	0.28
	(0.52)	(0.75)	(0.74)	(0.52)	(0.75)	(0.75)
Country of Origin = Turkey	0.18	0.19	− 0.14	− 0.93	− 0.51	− 0.73
	(0.45)	(0.59)	(0.57)	(0.63)	(0.87)	(0.84)
Country of Origin = Middle East/Northern Africa	0.14	− 0.14	− 0.18	0.18	− 0.11	− 0.15
	(0.43)	(0.52)	(0.51)	(0.43)	(0.53)	(0.51)
Religion = Islamic	2.91^***^	2.12^***^	2.32^***^	2.86^***^	2.11^***^	2.32^***^
	(0.41)	(0.56)	(0.54)	(0.41)	(0.56)	(0.54)
Attendance of religious services	0.19^**^	0.18	0.22^*^	0.09	0.13	0.19
	(0.10)	(0.13)	(0.13)	(0.10)	(0.14)	(0.14)
Country of Origin = Turkey * Attendance of religious services				0.63^**^	0.43	0.37
				(0.25)	(0.40)	(0.39)
Embeddedness differential	0.24^***^	0.20^***^	0.21^***^	0.24^***^	0.20^***^	0.21^***^
	(0.06)	(0.07)	(0.07)	(0.06)	(0.07)	(0.07)
Politics news on social media		− 0.09	− 0.09		− 0.08	− 0.08
		(0.12)	(0.12)		(0.12)	(0.12)
Network EMIP voting		1.00^***^			1.00^***^	
		(0.18)			(0.18)	
Partner votes EMIP			1.56^**^			1.55^**^
			(0.62)			(0.62)
Parents vote EMIP			1.97^***^			1.92^***^
			(0.56)			(0.57)
Friends vote EMIP			1.48^***^			1.49^***^
			(0.46)			(0.46)
R^2^	0.35	0.44	0.45	0.36	0.45	0.45
Num. obs.	422	224	233	422	224	233

^***^p < 0.01; ^**^p < 0.05; ^*^p < 0.1; Country of Origin reference is ‘other’

**Fig. 1:**
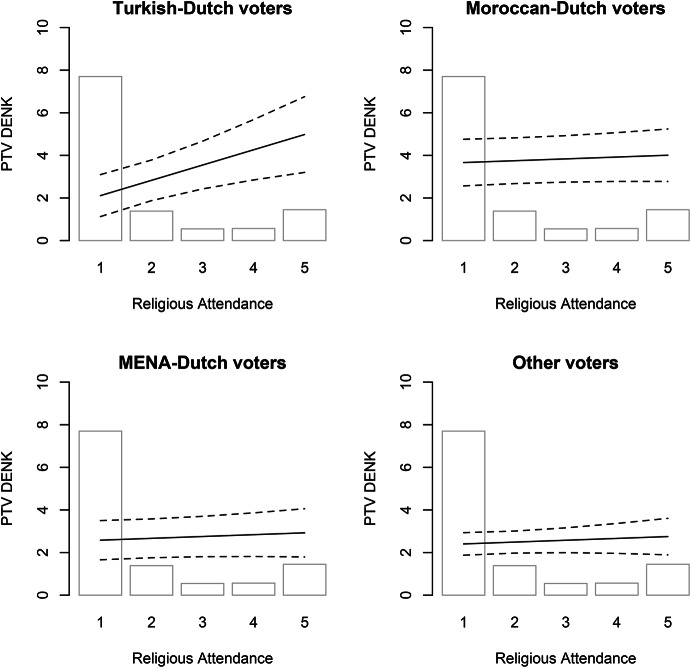
Ethnicity, Religious Attendance, and PTV DENK. Based on Model 2 (95% confidence interval; other variables at mean/median)

**Fig. 2:**
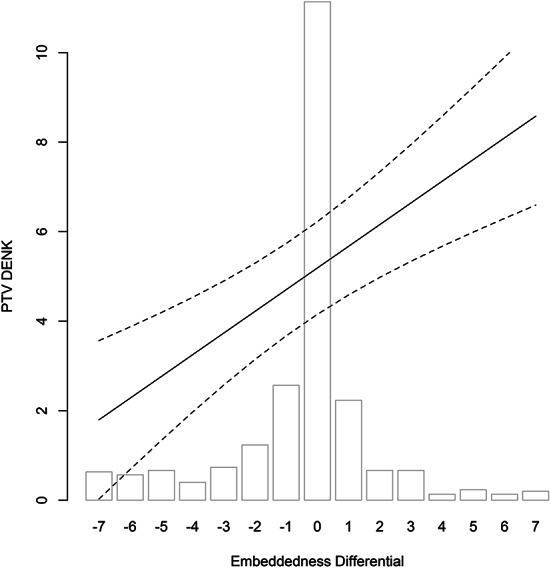
Embeddedness, Differential and PTV DENK. Based on Model 2 (95% confidence interval; other variables at mean/median)

**Fig. 3:**
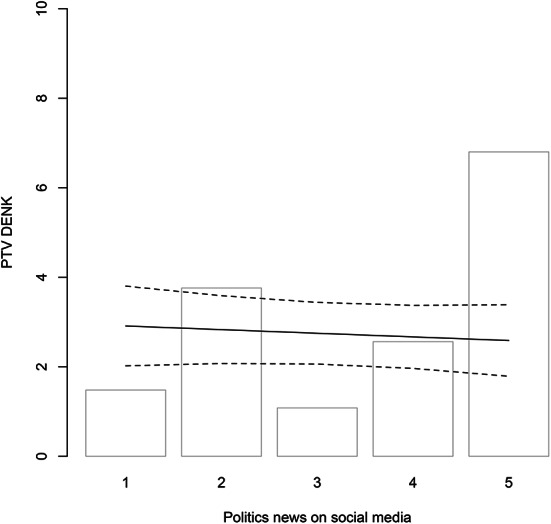
Social Media and PTV DENK DENK. Based on Model 5 (95% confidence interval; other variables at mean/median)

**Fig. 4:**
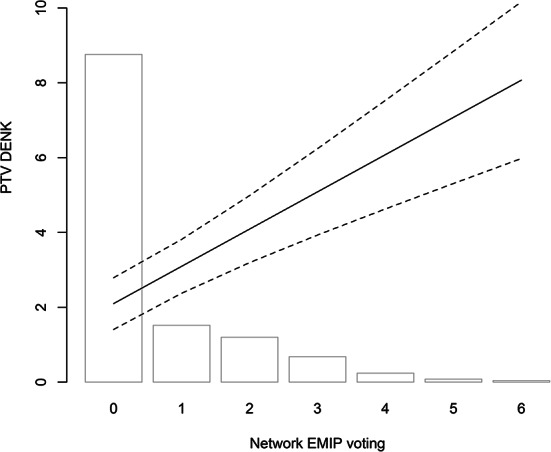
Network EMIP Voting and PTV DENK. Based on Model 5 (95% confidence interval; other variables at mean/median)

### Quantitative Results

Our key results are presented in Tables 1 and visualized in Figs. 1, 2, 3 and 4. The Appendix examines the robustness of these results and alternative specifications. Here, we discuss the main results per hypothesis and do not explore the control variables.^12^

The Religious Network Hypothesis concerns the importance of integration in ethno-religious organizations and in particular its effect on Turkish-Dutch citizens. We examine the effect of attendance of religious services.^13^ Model 1, which includes only variables from the first wave and therefore has the highest N, finds a clear positive effect: among those who attend religious services once a week or more, most have a PTV nearly one point higher than those who never do. The Appendix sustains these patterns. Model A4 shows that respondents who are members of a religious organization have a one-and-a-half points higher PTV for DENK than those who are not. Model 2 and 3 include variables from the first and second wave. In Model 3 the coefficient for attendance of religious services is not significant, while in the other it is. This likely due to the lower N but it is in the same and expected direction. This shows support for the Religious Network Hypothesis. 14

The Turkish-Dutch Religious Network Hypothesis specified that this effect is stronger for voters with a Turkish-Dutch background. Models 4–6 look into this, and Fig. 1 below visualizes Model 6. In Model 4, with only questions from the first wave and therefore the higher N, the effect of attendance of religious services is clearly stronger among respondents with a Turkish background. For them, the increase is three points on the PTV for DENK, compared to a non-significant 0.3-point effect found for other respondents. In the smaller-N models with the respondents that participated in both waves (Models 5–6), an effect of similar size is still present, but it is no longer statistically significant. These patterns persist if we add an additional control variable (Table A8) and restrict our sample to respondents from the Middle East and North Africa (Table A9).^15^ Overall, as expected, religious embeddedness is associated with a higher PTV for DENK, and most strongly so among Turkish-Dutch citizens.^16^

The Personal Network Hypothesis concerns the personal network of respondents. We examine the extent to which friends and family vote DENK or for another ethnic minority interest party, and whether respondents’ social circle mainly consists of people with the same ethnicity or of people without a migration background – voting in the personal network only being available in the post-election wave. Figure 2 shows the effect of social embeddedness. The independent variable is the difference between how often one sees co-ethnic friends and friends without a migration background. A higher value indicated a stronger embeddedness in an ethnically similar and homogenous group, compared to seeing friends without a migration background. The effect is clearly positive, as expected, with a maximum impact of more than three points on the PTV. This effect is quite consistent: in thirteen out of fourteen tests it is statistically significant (Models 1–6; A8; A16-A22).^17^

If we focus on how the respondents’ social and familial network vote, we see this pattern as well: Models 2 and 5 (and Fig. 4) show that for every acquaintance one has that votes for an ethnic minority interest party, the PTV to DENK increases by one. Employing relatively small samples, this effect is consistent: in all six models, the effect is significant and of equal size (Model A9; A13; A15; A17; A20). Additionally, Model A10 in the appendix focuses on three relationships (parents, partner, and friends): the effect of one’s parents is strongest, followed by one’s partner and one’s friends. The effect for these is also consistent: in only one model out of six, one of these coefficients is not significant (Models 3; 6; A10-A11; A18; A21).^18^ The Familial Network Hypothesis is supported by the strong impact of parents and partners.

The final hypothesis is the Online Network Hypothesis. If we examine indicators of the use of social media, (Models 2–3; 5–6; A6; A13; A15; A17-A18; A20-A21), we do not find a statistically significant effect. Figure 3 exemplifies this by visualizing the effect of consuming online political news in general. Before rejecting the hypothesis, we attempted to rule out alternative explanations for the absence of an effect (Table A11). For instance, it may be that DENK only has a prominent place in one’s feed if one’s connections are also more likely to vote DENK (and share their posts). If so, social media consumption is only likely to influence voting on DENK in combination with having such a network. Therefore, in Models A27-A29, we model the interaction between personal network and social media use and visualize them in Figures A1-A2. We examine having friends from the country of origin or EMIP voting among one’s network as a factor. This shows no positive significant interaction. In short: we find no evidence that activity on social media boosts the propensity to vote DENK.^19^ Integration into ethno-religious networks and personal networks seem more important.

## Conclusion

Our central goal was to determine how social networks affect voting for ethnic minority interest parties. Taking a broad and open social network perspective and applying this to the existing literature on voting behavior among ethnic minority citizens as well as sixteen in-depth interviews with Turkish-Dutch and Moroccan-Dutch citizens, we identified relatively nuanced and partially new views on three potentially relevant networks: religious networks, personal networks, and virtual networks.

For religious networks, we proposed that those who are more embedded in religious networks are more likely to vote for ethnic minority interest parties, and that this effect was stronger for Turkish-Dutch citizens. Our interviews and our statistical analyses show a marked difference between Moroccan and Turkish migrant communities. Turkish-Dutch mosques are an area for political discussion and mobilization; in the Moroccan-Dutch community, religion and politics are separated – a pattern that deserves comparative study. Considering the specific mechanisms for the religious network effects found, our quantitative analyses could however not distinguish between the different mechanisms, but the qualitative finding suggest that the area around mosques is particularly important as a site for campaigning and that political discussion takes place around the mosque leading to knowledge transfer. It does not seem to be the mosque leadership and their messages as such that matter, but the social infrastructure offered by mosques, which stresses the more general relevance of how an organization’s political role is seen for understanding the impact of any societal organization. As such our study links the U.S. and European literatures. White and Laird (2020) showed the importance of networks for voting among Black Americans, while Van der Zwan et al. (2020) and Vermeulen et al. (2020b) show that in Europe, the concentration of groups in certain areas matters for vote choice. In relatively homogenous ethno-religious networks, social norms and pressure to vote for a certain party can be prevalent, as long as the setting is politicized (as in the Turkish community).

For personal networks, we proposed that the extent to which family and friends voted for ethnic minority interest parties increased the chance of voting an ethnic minority interest party. We find strong evidence for the importance of familial voting, but also for the larger personal network. Part of this is also the ethnic composition of a respondents’ network apart from their voting: the more people with the same migration background and the fewer people without a migration background are part of this network, the more likely one is to vote for an ethnic minority interest party. Indirect experiences and shared grievances are less likely to come up in the study of dominant social groups. This might apply to other minority groups (e.g., LHBTQIA+, cf. Turnbull-Dugarte, 2020).

For virtual networks, we examine the effect of seeing political news online. While our interviews indicated that many Turkish-Dutch and Moroccan-Dutch respondents saw messages from the ethnic minority interest party in question online, this did not translate into a relationship between seeing political news online and voting DENK. Here, we should recall that in the qualitative analyses we found that almost *everyone* surveyed had come across DENK-related posts, independently of whether they voted for the party. The survey shows high levels of online political news consumption. It could not measure seeing DENK content specifically or via whom it reached respondents. Moreover, respondents might also have self-selected into following DENK if they prefer the party. Future, quantitative analysis on *what kind of posts* citizens see, from *what kind of messenger* may provide better tests of the patterns seen in the qualitative analyses. Our study adds to a growing understanding that the impact of social media is complex, and not a simple funnel to segregation for minority citizens.

We focused on voting for a particular party in a particular country. What do our results say beyond the border of our case and beyond the minority focused on? As Lubbers et al. (2023) observe, ethnic minority interest parties exist in seven other Western democracies, and ongoing patterns of migration and globalization suggest more to come. Previous studies on voting patterns for these parties have mainly focused on the policy views of respondents (Vermeulen et al., 2020a). However, our mixed-methods results clearly underscore the importance of personal, familial, and religious networks, but in ways that are not completely like what is known in the majority-population literature. Further study of the link between religion and politics, campaigning practices, groups norms, and identity-based grievances is necessary, beyond these particular minority groups. As these processes interweave with strong in-group ties while weakening cross-group linkage (i.e., segregation), they matter for democratic functioning in multicultural societies in general.

While our focus was on an ethnic minority *party*, the found mechanisms might also translate to party choice and preference voting for minority candidates in list systems, or minority contenders in primary elections in majoritarian systems (Farrer et al., 2018; Zuckerman et al., 1994). Indeed, our results substantiate Stevens and Bishin’s (2011) finding that the contact between political actors and citizens works differently for minorities. Understanding along which lines boundary-making is taking place is important. For instance, in the U.S. context, migrant groups are subject to different boundary-making processes than racial minorities, whereas for European Turkish and Moroccan communities there is a far stronger interlinkage between migration and racialization. The contextuality of boundary work matters for the specific mechanisms found in this study and can help to further develop our understanding of how different organizational and personal networks shape voting for specific minority candidates and parties. Thus, while the literature pays attention to social networks for voting, it is clearly seen as less important than policy positions. We believe that our paper shows the value of looking at the networks in which citizens operate for their voting behavior, particularly for ethnic, racial and other minority groups.

## Electronic Supplementary Material

Below is the link to the electronic supplementary material.

Supplementary Material 1

## Data Availability

The data and replication code for the quantitative study is available through the Political Behavior Dataverse via 10.7910/DVN/AIJVJB. Anonymized transcripts of the qualitative interviews are available upon request.

## Footnotes

1 Azabar et al. (2020) on preference voting and Vermeulen et al. (2020b) on bloc voting in Europe, and Stevens and Bishin (2011) on turnout and White and Laird (2020) on vote choice in the United States.

2 The lead author who has ample experience in interviewing and a background in Middle-Eastern studies.

3 Except Japan and Indonesia; including Turkey.

4 It correlates to actual vote across Europe (Van der Eijk et al., 2006).

5 16% answer ‘Don’t Know’ for DENK: 12% among Moroccan-Dutch, 9% among Turkish-Dutch, 19% for Dutch citizens with another MENA background.

6 Formulation of items is given in Table A3 in the Appendix.

7 Both variables do not assume that all respondents are Muslim and concern attendance of *any* religious service or membership in any religious organization. The Appendix (Table A5) examines the importance of Islamic religious orientation.

8 H = 0.35.

9 1 = male; 0 = female and non-binary.

10 1 = thirty or younger; 0 = older than thirty.

11 1 = degree from a university (including polytechnical institutes); 0 = without such a degree.

12 Of our controls, the effect of left–right distance stands out (Table A8). This has a clear but separate effect on voting for DENK. Respondents who place themselves closer to DENK give it a higher PTV.

13 Model A5 in Table A5 examines religious attendance and mosque membership together. Here only the former is significant.

14 The effect of attendance of religious services and membership of religious organizations is strong among Muslims, but absent among non-Muslims; moreover, the explanatory power of the model with the interaction effect of attendance for being Turkish is slightly higher than for being Muslim (Table A10).

15 Table A7 shows a significant interaction with embeddedness in religious networks for other MENA respondents, but not for Moroccan-Dutch respondents.

16 It is surprising that Moroccan-Dutch citizens are more likely to vote DENK (Model 1 and 4 in Table 1 and Appendix A5), which was not mentioned in the literature and was not visible in the interviews. Two possible explanations are: (1) that Moroccan-Dutch Azarkan led DENK instead of his Turkish-Dutch predecessor; (2) the category Turkish-Dutch in DEMES includes Dutch-Kurdish respondents which are less likely to vote for DENK.

17 Only in Model A20 (*N* = 142) is the effect non-significant. Table A5 shows that having co-ethnic friends and having friends without a migration background are separately correlated to voting DENK.

18 Only non-significant in Model A21 (*N* = 148).

19 Figure A2 on the effect of friends’ and family’s voting shows a negative effect, suggesting that spending more time on the internet weakens one’s direct social network’s effect.
