# Supplementary material for: The Role of Networks in Mobilization for Ethnic Minority Interest Parties
Source: Polit Behav. 2024 Nov 9;47(3):1253–74. doi: 10.1007/s11109-024-09988-w (PMC12397191; doi:10.1007/s11109-024-09988-w)
Supplement: Supplementary file 1 — Supplementary Material 1 [file 11109_2024_9988_MOESM1_ESM.docx]

*Appendix*

| **Table of Contents** | | | |
| --- | --- | --- | --- |
| Appendix A1: Qualitative Study | | p.2 |  |
|  | Table A1: List of Respondents | p.2 |  |
|  | Interview Guide | p.3 |  |
|  | Coding Scheme | p.5 |  |
|  | Table A2: Final Patterns | p.6 |  |
| Appendix A2: Quantitative Study | | p.8 |  |
|  | Table A3: Question wording | p.8 |  |
|  | Table A4: DEMES Descriptives | p.9 |  |
|  | Table A5: Models with controls and a single variable of interest | p.10 |  |
|  | Table A6: Models differentiating between networks | p.11 |  |
|  | Table A7: Models with Interactions for Turkish-Dutch, Moroccan-Dutch and other MENA background | p.12 |  |
|  | Table A8: Models with left-right dimension | p.13 |  |
|  | Table A9: Model limited to respondents with a MENA background | p.14 |  |
|  | Table A10: Models comparing Islamic and Turkish-Dutch interactions | p.15 |  |
|  | Table A11: Models with interactions between social network and social media | p.16 |  |
|  | Figure A1: Social Media, Embeddedness Differential & PTV DENK | p.17 |  |
|  | Figure A2: Social Media, Network EMIP voting & PTV DENK | p.17 |  |

**Appendix A1: Qualitative Study**

| *Table A1: List of Respondents* | | | | | | | | | |
| --- | --- | --- | --- | --- | --- | --- | --- | --- | --- |
| **#** | **Date** | **Length** | **Background** | **Education** | **Gender** | **Age** | **Religion** | **Vote**  **National**  **2021** | **Vote**  **Municipal**  **2022** |
| 1 | 14/4/2022 | 53:00 | Moroccan | WO | Female | 23 | Islamic | D66 | PvdA |
| 2 | 15/4/2022 | 28:22 | Moroccan | WO | Female | 20 | Islamic | DENK | DENK |
| 3 | 20/4/2022 | 24:57 | Moroccan | WO | Female | 21 | Islamic | GL | GL |
| 4 | 22/4/2022 | 31:24 | Moroccan | HBO | Male | 20 | Islamic | DENK | GL |
| 5 | 22/4/2022 | 30:33 | Moroccan | HBO | Female | 24 | Islamic | DENK | Not |
| 6 | 24/4/2022 | 13:44 | Moroccan | MBO | Male | 23 | Islamic | Not | DENK |
| 7 | 25/4/2022 | 31:52 | Turkish | HBO | Male | 28 | Islamic | D66 | DENK |
| 8 | 25/4/2022 | 24:15 | Turkish | WO | Male | 25 | Islamic | GL | CDA |
| 9 | 29/4/2022 | 15:22 | Turkish | MBO | Male | 23 | Islamic | DENK | CDA |
| 10 | 29/4/2022 | 28:09 | Moroccan | HBO | Female | 30 | Islamic | D66 | Not |
| 11 | 6/5/2022 | 35:25 | Turkish | WO | Male | 21 | Islamic | PvdA | D66 |
| 12 | 9/5/2022 | 22:16 | Turkish | HBO | Female | 24 | Islamic | SP | Independent Local Party |
| 13 | 12/5/2022 | 36:39 | Turkish | WO | Male | 21 | Islamic | DENK | Not |
| 14 | 22/5/2022 | 29:31 | Turkish | WO | Male | 21 | Islamic | DENK | DENK |
| 15 | 23/5/2022 | 24:23 | Turkish | HBO | Female | 21 | Islamic | DENK | Not |
| 16 | 30/5/2022 | 39:55 | Moroccan | WO | Male | 21 | Islamic | DENK | DENK |

MBO: Vocational college; HBO: University of Applied Science; WO: University.

**Interview Guide**

*Introduction:*

- Questions about their ethnic background, religiosity, place of residence, age.
- Questions about their political preference. Views on politics in general. How would they briefly describe their reasons for voting for DENK/[other party]?
- Did they vote for a different party before?

*Affinity & Group Interest:*

- Questions regarding the similarities between them and the candidates of DENK/[other party]. How important is this to them? Why?
  - What other similarities are important? Is it religion, ethnicity, general migration background, social class?
  - If they vote for DENK, do they have a preference for Azarkan or Kuzu? Why?
- Questions about the perception that DENK/[other party] effectively represents the interests of their community. Does the party also represent the interests of their group?
- Questions about thoughts within the group. Do other Moroccan-Dutch/Turkish-Dutch individuals also believe that DENK/[other party] represents their interests well?

*Social Capital:*

- Questions regarding the social interaction they have within the community. Do they often go to the mosque? Do they have a lot of contact with people at the mosque? Are they also members of other organizations, or do they often meet with a fixed group of people/friends?
- Questions regarding politics in the mosque/organization. Is politics regularly discussed in the mosque/organization/group? Only during election time? How would they describe such conversations? What is the general trend? Does the imam (or someone else) explicitly talk about it? What do they say?
  - Are there (prominent) individuals within the mosque/organization who are politically active? How do they express their political activism? Do they talk a lot about their political views and their party?
- Questions about politicians in the mosque. Do politicians visit the mosque/associations? What do they talk about? How do they address the audience? In what language? Which party do they belong to?
  - What is the story that the politicians come to tell? Does it resonate with them? And why?
  - Other than politicians, do people visit who talk about Dutch politics? What do they talk about?
- Is there any campaigning or flyer distribution in or around the mosque? Are there flyers on the table at the mosque, or are there posters hanging? Do all parties distribute flyers?
  - Are the people who distribute flyers in or around the mosque regular visitors of the mosque themselves? Are they acquaintances of the interviewee?
- Questions regarding online political mobilization. Do they often see political messages?
  - Do they often receive political messages from acquaintances within their social network? If so, through which social media platform do they receive these messages, and in what form? How would they describe the general trend of such messages, and do they relate to a specific party? And through what acquaintances do they see these messages (for example, through the mosque/organization)? Are they also encouraged to vote for a particular party or candidate? Are such messages mainly advertisements, or are they shared by others? Do they follow DENK/[other party] themselves? Are they familiar with videos from DENK or another party? Do they also receive these videos? Are these videos sometimes from another party?

*Representation:*

- Questions regarding feelings about representation. Do they think DENK is the only good representative for Moroccan-Dutch and Turkish-Dutch voters? Are other parties still capable of representing Moroccan-Dutch and Turkish-Dutch voters?
- Can they explain why they think DENK is popular among Moroccan-Dutch/Turkish-Dutch voters? What do they hear around them about this?
- Does DENK represent the Moroccan-Dutch/Turkish-Dutch community better than existing parties such as the PvdA? Why, what is the difference? Are there specific substantive/political issues in which DENK performs better? What would be examples? Do they believe that other left-wing parties are too progressive on certain issues like LGBTQ+ rights, abortion, etc., which goes against their religious values? How does this factor into their voting choice?
- Are these political issues frequently discussed in the mosque/organization/group? Is there often negative talk about parties other than DENK?
- If they vote for DENK, would they always vote for DENK? If they don't vote for DENK, would they possibly vote for DENK? What factors are important for this?
  - How important are racism and discrimination for them to vote for DENK/[other party]?

*Conclusion:*

- Are there more things they would like to discuss regarding politics in the Netherlands? Are there other things they would like to discuss regarding DENK or another political party? Are there any reasons for them to vote for DENK/[other party] that haven't been addressed in this interview?

**Coding Scheme**

*Political Discussions:*

- In the mosque:
  - Not discussed
  - Generally discussed
  - Specific party/mobilization
  - Turkish politics*
- In the migrant organization:
  - Not discussed
  - Generally discussed
  - Specific party/mobilization
  - Turkish politics*
- With friends, family, network:
  - Not discussed
  - Generally discussed
  - Specific party/mobilization
  - Turkish politics*

*Mobilization:*

- Mosque:
  - Campaign within the mosque
  - Campaign possible within the mosque
  - Campaign outside the mosque
  - No campaign in the mosque
- In the migrant organization:
  - Campaign within the organization
  - Campaign possible within the organization
  - No campaign in the mosque

*Social media*

- Politics on social media:
  - Not discussed
  - Generally discussed/diverse parties
  - For a specific party/mobilization

* Since Turkish national politics was a recurring topic during the interviews with the Dutch-Turkish respondents, this has been used as a subcode during the analysis.

| *Table A2: Final Patterns* | | | | | | |
| --- | --- | --- | --- | --- | --- | --- |
| **Migration background** | | | **Moroccan** | **Moroccan** | **Turkish** | **Turkish** |
| **Vote choice** | | | **DENK** | **Other** | **DENK** | **Other** |
| Political discussion | | |  |  |  |  |
|  | In the mosque | |  |  |  |  |
|  |  | Politics is not discussed in the mosque, and there is no campaigning | 2; 4; 5; 9; 16 | 1; 10 | - | - |
|  |  | Dutch politics is not discussed in the mosque, and there is no campaigning by Dutch political parties | - | - | 9 | 8; 12 |
|  |  | Politics is not discussed in the mosque because the mosque is a place to pray | 2; 4; 16 | 1; 10 | - | - |
|  |  | The mosque/imam remains neutral and does not express a political preference | 2; 5; 16 | 1; 10 | 13; 15 | 8 |
|  |  | Turkish politics discussed in mosque | - | - | 7; 9; 13; 14 | 8 |
|  | In migrant organization | |  |  |  |  |
|  |  | Politics is discussed within the non-religious migrant organization in general; different parties are discussed | 2; 5 | - | 14 | 11 |
|  | With friends, family, network | |  |  |  |  |
|  |  | Voting choice influenced by explanations from family members | 2 | 1; 10 | 9; 14 | 12 |
| Mobilization | | |  |  |  |  |
|  | In the mosque | |  |  |  |  |
|  |  | DENK possibly campaigned in mosque, but they did not experience it themselves | 2; 5 | 1; 3; 6 | 7; 15 | 8 |
|  |  | Another party than DENK campaigned in the mosque | - | - | 7 | - |
|  |  | DENK campaigned in the mosque | - | - | 13; 14 | - |
|  |  | DENK candidates are acquaintances from mosque | 16 | - | 13; 14 | - |
|  |  | Campaign is conducted outside the mosque, in the streets | 2; 4; 16 | 3; 10 | 7; 9; 13; 14; 15 | 12 |
|  | In the migrant organization | |  |  |  |  |
|  |  | Another party than DENK campaigned in the organization | - | - | 7 | - |
|  |  | DENK campaigned in the organization | - | - | 14 | - |
| Social media | | |  |  |  |  |
|  | Politics on social media | |  |  |  |  |
|  |  | Respondent saw a great diversity of political messages on social media | 2; 5 | 1; 3; 10 | 7; 9 | 8; 11; 12 |
|  |  | Respondent did not get to see political posts on social media from acquaintances in own network | 4 | 6 | 7 | 12 |
|  |  | Respondent saw (campaign) messages from DENK because they follow DENK themselves on social media | 4; 5; 16 |  | 7; 13 |  |
|  |  | Videos/messages of DENK were circulated by acquaintances | 5 | 1; 10 | 13; 14; 15 | 8 |
|  |  | An acquaintance is active in DENK and they shared campaigns for that party on social media |  |  | 13; 14 |  |
|  |  | Especially ads from DENK on social media, but also other parties | 16 |  | 15 |  |
|  |  | Respondent saw political posts about other parties on social media from acquaintances in own network | 2; 16 | 1; 3; 10 | 15 | 11 |

In the first stage of analysis, we read the transcripts through, and used open coding so that a wide range of different categories emerged. After reading the initial transcripts, we reduced the number of categories and arranged them based on general themes in the interviews. After establishing these themes, we used codes and related subcodes to track the overarching patterns in the answers given. We then placed excerpts from the interviews together based on the (sub-)codes, after which we collated the coded data according to themes, and arranged them according to the four groups studied, Moroccan-Dutch DENK voters, Turkish-Dutch DENK voters, Moroccan Dutch non-DENK voters and Turkish Dutch non-DENK voters. Finally, after analysing the (sub-)codes arranged per group, we extracted patterns that recurred in the coded data. This last step of tracing the patterns that we found in the (sub)codes has specifically been done to find those mechanisms behind the already determined (sub)codes. With this analysis we have been able to determine which patterns are relevant for voting or not voting for DENK.

**Appendix A2: Quantitative Study**

| *Table A3: Question wording* | |
| --- | --- |
| **Variable** | **Question wording** |
| Attendance of religious services | How often do you generally attend church, the mosque or religious services?  Five answer categories, from ‘(almost) never’ to ‘once a week or more’ |
| Member of church, mosque, synagogue | The following questions are about possible memberships and your willingness to take political action.  Are you a member of a: Church, mosque or synagogue. |
| Embeddedness in network without a migration background | Please think about all friends you have who live in the Netherlands. How often do you meet friends originally from the Netherlands.  With seven options ranging from “less than once a year” to “almost every day”. |
| Embeddedness in network from country of origin | Please think about all friends you have who live in the Netherlands. How often do you meet friends originally from the country of origin?  With seven options ranging from “less than once a year” to “almost every day”. |
| People in their family or friend network votes EMIP | Did someone from your network vote for one of following political parties?  My Parent[s], My Child[ren], my other family members, My friends, My co-workers and My neighbours. More than one answer is possible.  The answer option was the three EMIPs together: “DENK, BIJ1 or NIDA” For partner, we recode a more precise question to the same format. |
| Politics news on social media | How often so you see messages about politicians and/or current political affairs on your Facebook, Instagram, Twitter, WhatsApp and/or YouTube?  With five answer categories. |

| *Table A4: DEMES Descriptives* |  |  |  |  |  |  |
| --- | --- | --- | --- | --- | --- | --- |
| **Variable** | **Mean** | **Median** | **S.D.** | **Min.** | **Max.** | **N** |
| PTV for DENK | 4.03 | 3.00 | 3.34 | 1.00 | 100 | 566 |
| Gender = Male | 0.47 | - | - | 0.00 | 1.00 | 761 |
| Age ≤ 30 | 0.31 | - | - | 0.00 | 1.00 | 731 |
| Education = BA/MA | 0.42 | - | - | 0.00 | 1.00 | 680 |
| Country of Origin = Morocco | 0.14 | - | - | 0.00 | 1.00 | 735 |
| Country of Origin = Turkey | 0.17 | - | - | 0.00 | 1.00 | 735 |
| Country of Origin = Middle East/Northern Africa | 0.13 | - | - | 0.00 | 1.00 | 735 |
| Embeddedness in network without a migration background | 6.62 | 7.00 | 1.79 | 1.00 | 8.00 | 690 |
| Embeddedness in network from country of origin | 6.18 | 7.00 | 2.11 | 1.00 | 8.00 | 683 |
| Embeddedness differential | -0.47 | 0.00 | 2.30 | -7.00 | 7.00 | 666 |
| Partner votes EMIP | 0.08 | - | - | 0.00 | 1.00 | 360 |
| Parents vote EMIP | 0.11 | - | - | 0.00 | 1.00 | 358 |
| Children vote EMIP | 0.03 | - | - | 0.00 | 1.00 | 360 |
| Other family members vote EMIP | 0.14 | - | - | 0.00 | 1.00 | 354 |
| Friends vote EMIP | 0.16 | - | - | 0.00 | 1.00 | 359 |
| Colleagues vote EMIP | 0.06 | - | - | 0.00 | 1.00 | 353 |
| Neighbours vote EMIP | 0.02 | - | - | 0.00 | 1.00 | 360 |
| Network EMIP voting^a^ | 0.60 | 0.00 | 0.11 | 0.00 | 6.00 | 313 |
| Religion = Islamic | 0.33 | - | - | 0.00 | 1.00 | 666 |
| Attendance of religious services | 1.86 | 1.00 | 1.42 | 1.00 | 5.00 | 699 |
| Member of church, mosque, synagogue | 0.18 | - | - | 0.00 | 1.00 | 361 |
| Politics news on social media | 3.60 | 4.00 | 1.47 | 1.00 | 5.00 | 392 |
| Left-Right Distance to DENK | 2.15 | 2.00 | 2.13 | 0.00 | 9.00 | 336 |

^a^ H = 0.35

| *Table A5: Models with a single concept of interest* | | | | | | | | | | | | |  |
| --- | --- | --- | --- | --- | --- | --- | --- | --- | --- | --- | --- | --- | --- |
| **Model** | | **A1** | **A2** | **A3** | **A4** | **A5** | **A6** | **A7** | **A8** | **A9** | **A10** | **A11** | |
| Intercept | | 2.84^***^ | 2.39^***^ | 1.90^***^ | 1.88^***^ | 1.39^***^ | 1.86^***^ | 2.93^***^ | 2.60^***^ | 1.94^***^ | 2.01^***^ | 2.02^***^ | |
|  | | (0.17) | (0.26) | (0.30) | (0.34) | (0.41) | (0.49) | (0.64) | (0.26) | (0.34) | (0.34) | (0.33) | |
| Gender = Male | |  | -0.07 | -0.07 | 0.11 | 0.11 | -0.03 | -0.01 | -0.02 | 0.13 | 0.05 | 0.06 | |
|  | |  | (0.26) | (0.26) | (0.33) | (0.33) | (0.33) | (0.26) | (0.26) | (0.33) | (0.34) | (0.32) | |
| Age ≤ 30 | |  | 0.57^**^ | 0.52^*^ | 0.33 | 0.21 | 0.21 | 0.46^*^ | 0.46^*^ | 0.14 | 0.03 | -0.04 | |
|  | |  | (0.27) | (0.27) | (0.35) | (0.36) | (0.36) | (0.27) | (0.27) | (0.35) | (0.35) | (0.34) | |
| Education = BA/MA | |  | -0.22 | -0.16 | -0.12 | 0.06 | -0.12 | -0.04 | -0.04 | -0.35 | -0.37 | -0.23 | |
|  | |  | (0.26) | (0.26) | (0.33) | (0.34) | (0.33) | (0.26) | (0.26) | (0.33) | (0.33) | (0.32) | |
| Country of Origin = Morocco | | 4.07^***^ | 1.37^***^ | 1.42^***^ | 1.43^**^ | 1.69^**^ | 1.31^*^ | 1.10^**^ | 1.08^**^ | 1.01 | 0.78 | 0.60 | |
|  |  | (0.37) | (0.50) | (0.51) | (0.67) | (0.70) | (0.67) | (0.51) | (0.51) | (0.73) | (0.74) | (0.73) | |
| Country of Origin = Turkey | | 2.42^***^ | 0.27 | 0.40 | 0.42 | 0.46 | 0.16 | 0.12 | 0.12 | 0.74 | 0.71 | 0.24 | |
|  |  | (0.34) | (0.43) | (0.44) | (0.56) | (0.58) | (0.56) | (0.44) | (0.44) | (0.56) | (0.57) | (0.55) | |
| Country of Origin = Middle East/Northern Africa | | 0.80^**^ | 0.17 | 0.17 | -0.04 | -0.12 | -0.09 | 0.14 | 0.15 | 0.06 | 0.11 | 0.10 | |
|  |  | (0.40) | (0.43) | (0.43) | (0.53) | (0.53) | (0.53) | (0.43) | (0.43) | (0.52) | (0.52) | (0.51) | |
| Religion = Islamic | |  | 3.25^***^ | 2.93^***^ | 2.90^***^ | 2.77^***^ | 3.10^***^ | 3.16^***^ | 3.14^***^ | 2.07^***^ | 2.08^***^ | 2.48^***^ | |
|  | |  | (0.39) | (0.41) | (0.51) | (0.53) | (0.50) | (0.40) | (0.40) | (0.53) | (0.55) | (0.52) | |
| Attendance of religious services | |  |  | 0.27^***^ |  | 0.32^**^ |  |  |  |  |  |  | |
|  |  |  |  | (0.10) |  | (0.16) |  |  |  |  |  |  | |
| Member of church, mosque, synagogue | |  |  |  | 1.48^***^ | 0.78 |  |  |  |  |  |  | |
|  |  |  |  |  | (0.41) | (0.53) |  |  |  |  |  |  | |
| Politics news on social media | |  |  |  |  |  | 0.12 |  |  |  |  |  | |
|  |  |  |  |  |  |  | (0.12) |  |  |  |  |  | |
| Embeddedness in network without a migration background | |  |  |  |  |  |  | -0.28^***^ |  |  |  |  | |
|  |  |  |  |  |  |  |  | (0.08) |  |  |  |  | |
| Embeddedness in network from country of origin | |  |  |  |  |  |  | 0.23^***^ |  |  |  |  | |
|  |  |  |  |  |  |  |  | (0.07) |  |  |  |  | |
| Embeddedness differential | |  |  |  |  |  |  |  | 0.25^***^ |  |  |  | |
|  |  |  |  |  |  |  |  |  | (0.06) |  |  |  | |
| Network EMIP voting | |  |  |  |  |  |  |  |  | 1.02^***^ |  |  | |
|  |  |  |  |  |  |  |  |  |  | (0.17) |  |  | |
| Partner votes EMIP | |  |  |  |  |  |  |  |  |  | 1.56^**^ | 1.63^***^ | |
|  | |  |  |  |  |  |  |  |  |  | (0.64) | (0.63) | |
| Parents vote EMIP | |  |  |  |  |  |  |  |  |  | 1.92^***^ | 2.00^***^ | |
|  | |  |  |  |  |  |  |  |  |  | (0.67) | (0.53) | |
| Children vote EMIP | |  |  |  |  |  |  |  |  |  | 0.11 |  | |
|  | |  |  |  |  |  |  |  |  |  | (1.11) |  | |
| Other family members vote EMIP | |  |  |  |  |  |  |  |  |  | 0.67 |  | |
|  |  |  |  |  |  |  |  |  |  |  | (0.64) |  | |
| Friends vote EMIP | |  |  |  |  |  |  |  |  |  | 1.24^**^ | 1.43^***^ | |
|  | |  |  |  |  |  |  |  |  |  | (0.53) | (0.46) | |
| Colleagues vote EMIP | |  |  |  |  |  |  |  |  |  | -0.11 |  | |
|  |  |  |  |  |  |  |  |  |  |  | (0.73) |  | |
| Neighbours vote EMIP | |  |  |  |  |  |  |  |  |  | 0.37 |  | |
|  |  |  |  |  |  |  |  |  |  |  | (1.24) |  | |
| R^2^ | | 0.21 | 0.32 | 0.33 | 0.32 | 0.34 | 0.29 | 0.35 | 0.35 | 0.43 | 0.44 | 0.42 | |
| Num. obs. | | 552 | 458 | 444 | 271 | 264 | 284 | 435 | 435 | 238 | 238 | 249 | |
|  | ^***^p < 0.01; ^**^p < 0.05; ^*^p < 0.1 | | | | | | | | | | | |  |

| *Table A6: Models differentiating between networks* | | | | | |
| --- | --- | --- | --- | --- | --- |
| **Model** | **A12** | **A13** | **A14** | **A15** |  |
| Intercept | 2.43^***^ | 3.98^***^ | 2.63^***^ | 4.00^***^ |  |
|  | (0.66) | (0.90) | (0.67) | (0.90) |  |
| Gender = Male | -0.01 | 0.12 | -0.06 | 0.09 |  |
|  | (0.26) | (0.33) | (0.26) | (0.34) |  |
| Age ≤ 30 | 0.41 | 0.01 | 0.43 | 0.00 |  |
|  | (0.28) | (0.36) | (0.27) | (0.36) |  |
| Education = BA/MA | -0.01 | -0.02 | 0.03 | 0.01 |  |
|  | (0.27) | (0.34) | (0.26) | (0.34) |  |
| Country of Origin = Morocco | 1.13^**^ | 0.65 | 1.27^**^ | 0.69 |  |
|  | (0.52) | (0.74) | (0.52) | (0.75) |  |
| Country of Origin = Turkey | 0.19 | 0.23 | -0.92 | -0.41 |  |
|  | (0.45) | (0.59) | (0.63) | (0.86) |  |
| Country of Origin = Middle East/Northern Africa | 0.13 | -0.15 | 0.17 | -0.12 |  |
|  | (0.43) | (0.52) | (0.43) | (0.52) |  |
| Religion = Islamic | 2.92^***^ | 2.19^***^ | 2.87^***^ | 2.18^***^ |  |
|  | (0.42) | (0.55) | (0.41) | (0.55) |  |
| Attendance of religious services | 0.19^**^ | 0.21 | 0.09 | 0.17 |  |
|  | (0.10) | (0.13) | (0.10) | (0.14) |  |
| Embeddedness in network without a migration background | -0.26^***^ | -0.42^***^ | -0.26^***^ | -0.41^***^ |  |
|  | (0.08) | (0.11) | (0.08) | (0.11) |  |
| Embeddedness in network from country of origin | 0.23^***^ | 0.09 | 0.23^***^ | 0.09 |  |
|  | (0.07) | (0.08) | (0.07) | (0.08) |  |
| Country of Origin = Turkey * Attendance of religious services |  |  | 0.63^**^ | 0.40 |  |
|  |  |  | (0.25) | (0.39) |  |
| Politics news on social media |  | -0.02 |  | -0.01 |  |
|  |  | (0.13) |  | (0.13) |  |
| Network EMIP voting |  | 1.02^***^ |  | 1.02^***^ |  |
|  |  | (0.18) |  | (0.18) |  |
| R^2^ | 0.35 | 0.46 | 0.36 | 0.46 |  |
| Num. obs. | 422 | 224 | 422 | 224 |  |
| ^***^p < 0.01; ^**^p < 0.05; ^*^p < 0.1 | | | | | |

| *Table A7: Models with Interactions for Turkish-Dutch, Moroccan-Dutch and other MENA background* | | | |  |
| --- | --- | --- | --- | --- |
| **Model** | **A16** | **A17** | **A18** | |
| Intercept | 2.54^***^ | 2.31^***^ | 2.26^***^ | |
|  | (0.35) | (0.62) | (0.61) | |
| Gender = Male | -0.09 | 0.05 | 0.04 | |
|  | (0.26) | (0.35) | (0.34) | |
| Age ≤ 30 | 0.44 | 0.02 | -0.14 | |
|  | (0.27) | (0.36) | (0.36) | |
| Education = BA/MA | 0.06 | 0.03 | 0.12 | |
|  | (0.26) | (0.35) | (0.34) | |
| Country of Origin = Morocco | 1.45^*^ | 0.56 | 0.49 | |
|  | (0.78) | (1.09) | (1.08) | |
| Country of Origin = Turkey | -1.03 | -0.61 | -0.77 | |
|  | (0.64) | (0.89) | (0.87) | |
| Country of Origin = Middle East/Northern Africa | -0.73 | -0.60 | -0.50 | |
|  | (0.67) | (0.86) | (0.84) | |
| Religion = Islamic | 2.81^***^ | 2.10^***^ | 2.32^***^ | |
|  | (0.41) | (0.56) | (0.54) | |
| Attendance of religious services | 0.01 | 0.07 | 0.16 | |
|  | (0.13) | (0.18) | (0.17) | |
| Country of Origin = Morocco * Attendance of religious services | -0.03 | 0.03 | -0.08 | |
|  | (0.25) | (0.38) | (0.38) | |
| Country of Origin = Turkey * Attendance of religious services | 0.71^***^ | 0.51 | 0.40 | |
|  | (0.26) | (0.42) | (0.41) | |
| Country of Origin = Middle East/Northern Africa * Attendance of religious services | 0.50^*^ | 0.24 | 0.16 | |
|  | (0.29) | (0.33) | (0.33) | |
| Embeddedness differential | 0.24^***^ | 0.20^***^ | 0.21^***^ | |
|  | (0.06) | (0.07) | (0.07) | |
| Politics news on social media |  | -0.07 | -0.08 | |
|  |  | (0.13) | (0.12) | |
| Network EMIP voting |  | 0.98^***^ |  | |
|  |  | (0.18) |  | |
| Partner votes EMIP |  |  | 1.52^**^ | |
|  |  |  | (0.63) | |
| Parents vote EMIP |  |  | 1.92^***^ | |
|  |  |  | (0.58) | |
| Friends vote EMIP |  |  | 1.49^***^ | |
|  |  |  | (0.47) | |
| R^2^ | 0.37 | 0.45 | 0.46 | |
| Num. obs. | 422 | 224 | 233 | |
| ^***^p < 0.01; ^**^p < 0.05; ^*^p < 0.1 | | | |  |

| *Table A8: Models with left-right dimension* | | | |  |
| --- | --- | --- | --- | --- |
| **Model** | **A19** | **A20** | **A21** | |
| Intercept | 4.04^***^ | 3.37^***^ | 3.48^***^ | |
|  | (0.49) | (0.80) | (0.81) | |
| Gender = Male | -0.01 | -0.03 | 0.03 | |
|  | (0.34) | (0.42) | (0.41) | |
| Age ≤ 30 | 0.02 | -0.41 | -0.44 | |
|  | (0.36) | (0.47) | (0.45) | |
| Education = BA/MA | -0.16 | -0.05 | -0.05 | |
|  | (0.34) | (0.42) | (0.41) | |
| Country of Origin = Morocco | 0.42 | 0.47 | 0.11 | |
|  | (0.65) | (0.88) | (0.88) | |
| Country of Origin = Turkey | -2.17^**^ | -1.23 | -1.44 | |
|  | (0.88) | (1.03) | (1.03) | |
| Country of Origin = Middle East/Northern Africa | 0.07 | 0.13 | 0.09 | |
|  | (0.60) | (0.69) | (0.66) | |
| Religion = Islamic | 2.92^***^ | 1.94^***^ | 2.44^***^ | |
|  | (0.55) | (0.71) | (0.68) | |
| Attendance of religious services | 0.07 | 0.13 | 0.17 | |
|  | (0.14) | (0.18) | (0.17) | |
| Left-Right Distance | -0.43^***^ | -0.39^***^ | -0.40^***^ | |
|  | (0.08) | (0.10) | (0.10) | |
| Country of Origin = Turkey * Attendance of religious services | 1.08^***^ | 0.70^*^ | 0.59 | |
|  | (0.33) | (0.42) | (0.43) | |
| Embeddedness differential | 0.18^**^ | 0.15 | 0.17^*^ | |
|  | (0.08) | (0.10) | (0.10) | |
| Politics news on social media |  | -0.03 | -0.06 | |
|  |  | (0.16) | (0.16) | |
| Network EMIP voting |  | 0.98^***^ |  | |
|  |  | (0.21) |  | |
| Partner votes EMIP |  |  | 0.80 | |
|  |  |  | (0.66) | |
| Parents vote EMIP |  |  | 1.85^***^ | |
|  |  |  | (0.67) | |
| Friends vote EMIP |  |  | 1.64^***^ | |
|  |  |  | (0.53) | |
| R^2^ | 0.41 | 0.54 | 0.54 | |
| Num. obs. | 251 | 142 | 148 | |
| ^***^p < 0.01; ^**^p < 0.05; ^*^p < 0.1 | | | |  |

| *Table A9: Model limited to respondents with a MENA background* | |
| --- | --- |
| **Model** | **A22** |
| Intercept | 3.10^***^ |
|  | (0.69) |
| Gender = Male | -0.35 |
|  | (0.44) |
| Age ≤ 30 | 0.06 |
|  | (0.46) |
| Education = BA/MA | -0.18 |
|  | (0.43) |
| Country of Origin = Morocco | 0.68 |
|  | (0.65) |
| Country of Origin = Turkey | -1.24 |
|  | (0.81) |
| Religion = Islamic | 2.89^***^ |
|  | (0.54) |
| Attendance of religious services | 0.16 |
|  | (0.19) |
| Country of Origin = Turkey * Attendance of religious services | 0.53^*^ |
|  | (0.30) |
| Embeddedness differential | 0.45^***^ |
|  | (0.12) |
| R^2^ | 0.38 |
| Num. obs. | 188 |
| ^***^p < 0.01; ^**^p < 0.05; ^*^p < 0.1 | |

| *Table A10: Models comparing Islamic and Turkish-Dutch interactions* | | | | |
| --- | --- | --- | --- | --- |
| **Model** | **A23** | **A24** | **A25** | **A26** |
| Intercept | 2.23^***^ | 2.04^***^ | 2.08^***^ | 1.98^***^ |
|  | (0.34) | (0.35) | (0.31) | (0.34) |
| Gender = Male | -0.18 | 0.04 | -0.13 | 0.06 |
|  | (0.26) | (0.33) | (0.26) | (0.33) |
| Age ≤ 30 | 0.52^*^ | 0.27 | 0.53^*^ | 0.30 |
|  | (0.27) | (0.35) | (0.27) | (0.35) |
| Education = BA/MA | -0.17 | -0.11 | -0.13 | -0.09 |
|  | (0.26) | (0.33) | (0.26) | (0.33) |
| Country of Origin = Morocco | 1.29^**^ | 1.30^*^ | 1.55^***^ | 1.55^**^ |
|  | (0.51) | (0.67) | (0.51) | (0.67) |
| Country of Origin = Turkey | 0.45 | 0.49 | -0.67 | 0.01 |
|  | (0.44) | (0.56) | (0.62) | (0.58) |
| Country of Origin = Middle East/Northern Africa | 0.17 | 0.06 | 0.21 | -0.03 |
|  | (0.43) | (0.53) | (0.43) | (0.53) |
| Religion = Islamic | 2.09^***^ | 2.44^***^ | 2.89^***^ | 2.87^***^ |
|  | (0.56) | (0.54) | (0.40) | (0.51) |
| Attendance of religious services | 0.10 |  | 0.17^*^ |  |
|  | (0.13) |  | (0.10) |  |
| Religion = Islamic * Attendance of religious services | 0.42^**^ |  |  |  |
|  | (0.20) |  |  |  |
| Member of church, mosque, synagogue |  | 0.74 |  | 1.03^**^ |
|  |  | (0.52) |  | (0.45) |
| Religion = Islamic * Member of church, mosque, synagogue |  | 1.96^**^ |  |  |
|  |  | (0.85) |  |  |
| Country of Origin = Turkey * Attendance of religious services |  |  | 0.62^**^ |  |
|  |  |  | (0.25) |  |
| Country of Origin = Turkey * Member of church, mosque, synagogue |  |  |  | 2.56^**^ |
|  |  |  |  | (1.07) |
| R^2^ | 0.33 | 0.34 | 0.34 | 0.34 |
| Num. obs. | 444 | 271 | 444 | 271 |
| ^***^p < 0.01; ^**^p < 0.05; ^*^p < 0.1 | | | | |

| *Table A11: Models with interactions between social network and social media* | | | |
| --- | --- | --- | --- |
| **Model** | **A27** | **A28** | **A29** |
| Intercept | 2.78 | 2.31^***^ | 2.35^***^ |
|  | (2.08) | (0.59) | (0.57) |
| Gender = Male | 0.19 | 0.18 | 0.17 |
|  | (0.33) | (0.34) | (0.33) |
| Age ≤ 30 | 0.09 | 0.08 | -0.10 |
|  | (0.35) | (0.36) | (0.35) |
| Education = BA/MA | -0.08 | -0.07 | 0.01 |
|  | (0.34) | (0.34) | (0.33) |
| Country of Origin = Morocco | 0.76 | 0.70 | 0.39 |
|  | (0.74) | (0.74) | (0.73) |
| Country of Origin = Turkey | 0.38 | 0.41 | 0.06 |
|  | (0.57) | (0.58) | (0.56) |
| Country of Origin = Middle East/Northern Africa | -0.16 | -0.14 | -0.09 |
|  | (0.52) | (0.53) | (0.52) |
| Religion = Islamic | 2.05^***^ | 1.92^***^ | 1.97^***^ |
|  | (0.55) | (0.56) | (0.54) |
| Politics news on social media | 0.42 | -0.05 | -0.03 |
|  | (0.56) | (0.14) | (0.14) |
| Embeddedness in network without a migration background | -0.30 |  |  |
|  | (0.28) |  |  |
| Embeddedness in network from country of origin | 0.19 |  |  |
|  | (0.21) |  |  |
| Network EMIP voting | 2.07^***^ | 2.11^***^ |  |
|  | (0.68) | (0.69) |  |
| Embeddedness differential |  | 0.26 | 0.27 |
|  |  | (0.18) | (0.18) |
| Partner votes EMIP |  |  | 4.97^**^ |
|  |  |  | (2.43) |
| Parents vote EMIP |  |  | 2.55 |
|  |  |  | (2.10) |
| Friends vote EMIP |  |  | 5.96^***^ |
|  |  |  | (2.12) |
| Politics news on social media * Embeddedness in network without a migration background | -0.04 |  |  |
|  | (0.08) |  |  |
| Politics news on social media * Embeddedness in network from country of origin | -0.03 |  |  |
|  | (0.05) |  |  |
| Politics news on social media * Network EMIP voting | -0.22 | -0.24 |  |
|  | (0.15) | (0.15) |  |
| Politics news on social media * Embeddedness differential |  | -0.01 | -0.02 |
|  |  | (0.05) | (0.05) |
| Politics news on social media * Partner votes EMIP |  |  | -0.79 |
|  |  |  | (0.55) |
| Politics news on social media * Parents vote EMIP |  |  | -0.02 |
|  |  |  | (0.46) |
| Politics news on social media * Friends vote EMIP |  |  | -0.99^**^ |
|  |  |  | (0.47) |
| R^2^ | 0.46 | 0.45 | 0.46 |
| Num. obs. | 227 | 227 | 236 |
| ^***^p < 0.01; ^**^p < 0.05; ^*^p < 0.1 | | | |

| *Figure A1: Social Media, Embeddedness Differential & PTV DENK* | *Figure A2: Social Media, Network EMIP voting & PTV DENK* |
| --- | --- |
|  |  |
| Based on Model A27 with a 95% confidence interval; Grey line = 10% lowest value for differential; Black line = 90% highest value for differential; other variables at mean/median. | Based on Model A27 with a 95% confidence interval; Grey line = 10% lowest value for differential; Black line = 90% highest value for differential; other variables at mean/median. |
